# Flowability of Gel-Matrix and Magnetorheological Response for Carrageenan Magnetic Hydrogels

**DOI:** 10.3390/gels7020056

**Published:** 2021-05-06

**Authors:** Junko Ikeda, Tomoki Kurihara, Keiju Ogura, Shota Akama, Mika Kawai, Tetsu Mitsumata

**Affiliations:** Graduate School of Science and Technology, Niigata University, Niigata 950-2181, Japan; f17k501b@mail.cc.niigata-u.ac.jp (J.I.); kuri.tomokies@gmail.com (T.K.); t17g428d@mail.cc.niigata-u.ac.jp (K.O.); aaa.rock@icloud.com (S.A.); mikagoro@eng.niigata-u.ac.jp (M.K.)

**Keywords:** stimuli-responsive material, magnetic gel, viscoelastic property, magnetorheologylogy, carrageenan

## Abstract

The relationship between rheological features in the absence of a magnetic field and magnetic response was investigated for κ-carrageenan magnetic hydrogels containing carbonyl iron particles. The concentration of carrageenan was varied from 1.0 to 5.0 wt%, while the concentration of carbonyl iron was kept at 70 wt%. The magnetic response revealed that the change in storage modulus Δ*G′* decreased inversely proportional to the carrageenan concentration. A characteristic strain γ_1_ where *G*′ equals to *G*″ was seen in a strain range of 10^−3^. It was found that Δ*G*′ was inversely proportional to the characteristic stress at γ_1_. Another characteristic strain γ_2_ where the loss tangent significantly increased was also analyzed. Similar to the behavior of γ_1_, Δ*G*′ was inversely proportional to γ_2_. The characteristic stresses at γ_1_ and γ_2_ were distributed at 80–720 Pa and 40–310 Pa, respectively. It was revealed that a giant magnetorheology higher than 1 MPa can be observed when the characteristic stresses at γ_1_ and γ_2_ are below approximately 240 Pa and 110 Pa, respectively.

## 1. Introduction

Magnetic soft material consisting of polymeric matrix and magnetic particles demonstrates drastic changes in physical properties in response to magnetic fields, and it has great potential in various applications [1,2,3]. To achieve a high and fast response of the elasticity change, a variety of improvements have been reported [4,5,6,7,8,9]. Under a gradient magnetic field, magnetic soft material undergoes elongation behavior proportionally to the field gradient [10]. Under a uniform magnetic field, the magnetorheological effect (MR effect) is observed, where the viscoelastic properties vary in response to the magnetic field. The MR effect is basically caused by an anisotropic structure of magnetic particles formed in a matrix of a cross-linked polymer, which is called a chain structure. The structure can be observed by an optical microscopy when the concentration of magnetic particles is low [11]. Gundermann and Odenbach investigated the movement of magnetic particles for a magnetic elastomer with magnetic particles of 2 wt% using an X-ray computed tomography (CT). They found that the magnetic particles move and form a chain-like structure in the cross-linked matrix [12]. We also found by an X-ray CT with high resolution that magnetic particles make an anisotropic structure under a magnetic field [13]. When forming the chain structure, the polymer network should be destroyed in local owing to the relative movement of magnetic particles. Actually, an indication has been observed by SEM observation that a carrageenan network was stretched by magnetic particles [14]. If the polymer network is easy to deform or elongate, magnetic particles easily align in the direction of magnetic field, resulting in high and fast magnetic response. Of course, the increase in storage modulus by the structuring of magnetic particles is far higher than the decrease in the modulus due to the breaking of cross-linking point; therefore, the modulus change being observed is normally positive.

Magnetic hydrogels made of polysaccharides have been widely investigated as a drug carrier [15,16,17,18,19,20,21,22]. On the other hand, they are known for demonstrating a significant MR effect compared with magneto-responsive gels or elastomers made of synthetic polymers [23,24,25,26]. That is, the storage modulus for polysaccharide magnetic hydrogels is dramatically increased by a magnetic field even at high off-field modulus. As we reported briefly [24], this is a feature commonly observed in polysaccharide magnetic hydrogels. As a possible reason for this, we presented a hypothesis that there is a special structure enabling the high mobility of magnetic particles in polysaccharide hydrogels. However, the details relating to the special structure for polysaccharide magnetic hydrogels could not be elucidated in the paper. We focused here on the viscoelastic property of carrageenan magnetic hydrogels and investigated the flowability of magnetic particles in carrageenan gel by measuring the dynamic modulus. Polysaccharide hydrogels are formed by a weak physical bond between polysaccharide chains [27]. Therefore, the physical bond should be easily destructed by weak stress and, vice versa, it is easily reconstituted by self-healing [28,29,30]. In this study, we discuss the relationship between the flowability of the gel-matrix and the MR amplitude (the change in storage modulus).

## 2. Results and Discussion

**Figure 1:**
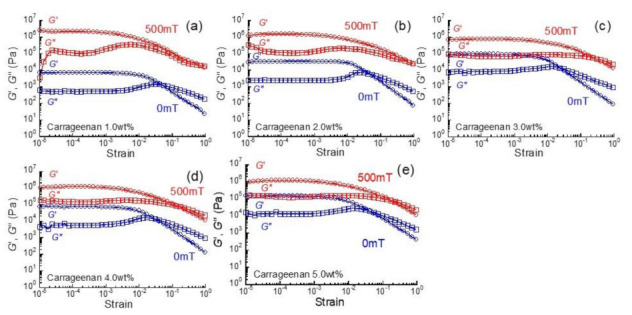
Strain dependence of storage modulus and loss modulus at 0 mT and 500 mT for magnetic hydrogels with various carrageenan concentrations (carbonyl iron (CI): 70 wt%, *f* = 1 Hz). (**a**) 1.0 wt%, (**b**) 2.0 wt%, (**c**) 3.0 wt%, (**d**) 4.0 wt%, (**e**) 5.0 wt%.

Figure 1 displays the strain dependence of storage modulus and loss modulus at 0 mT and 500 mT for magnetic hydrogels with various carrageenan concentrations. The storage modulus at a strain of 10^−4^ for 1 wt% carrageenan was 7.4 × 10^3^ Pa at 0 mT and 2.2 × 10^6^ Pa at 500 mT, which was in good agreement with our previous results [14,23,24]. At 0 mT, a plateau region of *G*′ or *G*″ was observed at strains below 10^−3^ for all magnetic hydrogels showing linear viscoelasticity. At large strains, *G*′ decreased remarkably with the strain, indicating that the cross-linking point was destructed by the large strains. The *G*″ first increased at a strain around 10^−2^, then took a maximum and decreased, which has been classified as type Ⅲ flow (weak strain overshoot) classified by Hyun et al. [31]. At a strain slightly higher than the maximum, *G*′ crossed with *G*″, which indicates that the gel matrix changes from solid-like to liquid-like.

**Figure 2:**
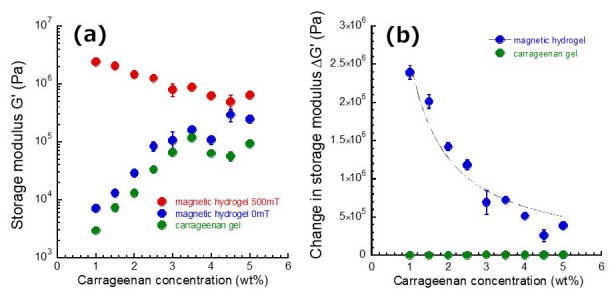
Carrageenan concentration dependence of (**a**) storage modulus at 0 and 500 mT and (**b**) change in storage modulus at 500 mT for magnetic hydrogels (CI: 70 wt%, *f* = 1 Hz) and carrageenan hydrogels without magnetic particles.

Figure 2a depicts the relationship between the storage modulus and carrageenan concentration for magnetic hydrogels. At 0 mT, the storage modulus for magnetic hydrogels took the lowest value at 1.0 wt%, and increased with the carrageenan concentration. The storage modulus was almost constant at concentrations above 3.0 wt%. The storage modulus for carrageenan hydrogels without magnetic particles is also shown in the figure, and it showed a similar trend with magnetic hydrogels. At 500 mT, the storage modulus took the maximum value at 1.0 wt% and gradually decreased with the carrageenan concentration. Similar to the off-field modulus, the on-field modulus was constant at concentrations above 3.0 wt%. It can be considered that a further decrease in the mobility of magnetic particle does not occur at above the concentration.

Figure 2b demonstrates the relationship between the change in storage modulus at 500 mT and carrageenan concentration for magnetic hydrogels. Because the on-field modulus is one order of magnitude higher than the off-field modulus, the change in storage modulus Δ*G*′ was inversely proportional to the carrageenan concentration at whole concentrations, as indicated by the dotted line in the figure. It has been widely accepted that the increase in the elastic modulus by magnetic field originates from the chain-like formation of magnetic particles. Actually, the behavior of magnetic particles within matrices of cross-linked polymer has been cleared by μ-CT observation [12,13]. It can be considered that the mobility of magnetic particles largely decreased with increasing of the carrageenan concentration owing to a dense network of carrageenan chains.

**Figure 3:**
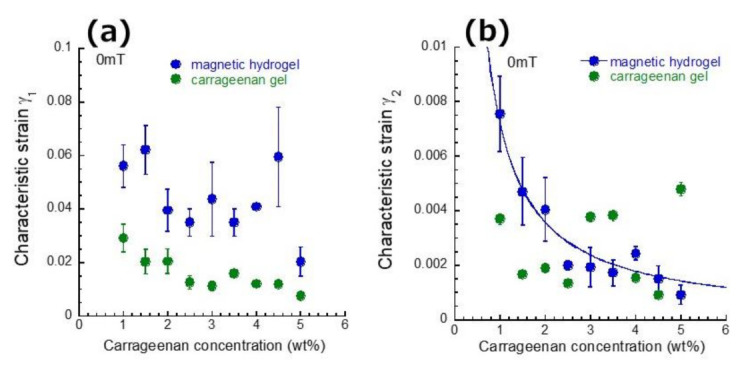
Relationship between characteristic strain (**a**) γ_1_ or (**b**) γ_2_ and carrageenan concentration for magnetic hydrogels (CI: 70 wt%) and carrageenan hydrogels without magnetic particles.

Figure 3a shows the relationship between the characteristic strain γ_1_ at 0 mT and carrageenan concentration for magnetic hydrogels. The characteristic strain γ_1_ was determined by the strain where *G*′ equals to *G*″ in Figure 1. The γ_1_ for magnetic hydrogels was almost independent of the carrageenan concentration, although it has a trend to decrease with the carrageenan concentration. Interestingly, this means that a physical bond of the carrageenan network becomes easier to break with increasing of the carrageenan concentration. A similar trend of strain softening was also seen in γ_1_ for carrageenan hydrogels without magnetic particles. Therefore, this is an intrinsic property for carrageen gel. The absolute values of γ_1_ for carrageenan hydrogels without magnetic particles were approximately 1/4 of those for magnetic hydrogels, suggesting that the carrageenan network became tough by incorporating with magnetic particles.

Figure 3b demonstrates the relationship between the characteristic strain γ_2_ and carrageenan concentration for magnetic hydrogels. Clearly, γ_2_ for magnetic hydrogels decreased inversely proportional to the carrageenan concentration. On the other hand, γ_2_ for carrageenan hydrogels was almost independent of the carrageenan concentration.

**Figure 4:**
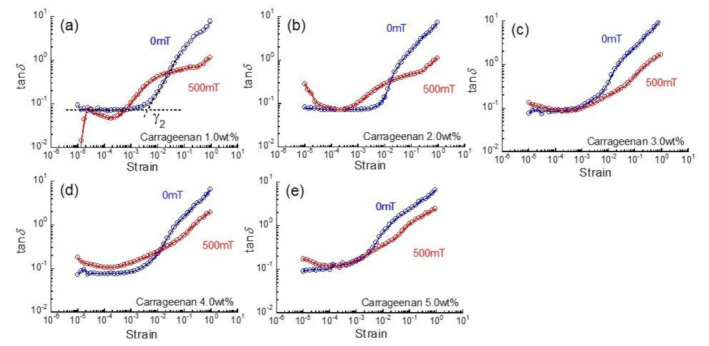
Strain dependence of loss tangent at 0 and 500 mT for magnetic hydrogels with various carrageenan concentrations (CI: 70 wt%, *f* = 1 Hz), (**a**) 1.0 wt%, (**b**) 2.0 wt%, (**c**) 3.0 wt%, (**d**) 4.0 wt%, (**e**) 5.0 wt%.

Figure 4 displays the strain dependence of loss tangent for magnetic hydrogels with various carrageenan concentrations. At 0 mT, the loss tangent was almost constant at strains below 10^−3^ and sharply increased at a certain strain, which is called a characteristic strain γ_2_ hereafter. The characteristic strain γ_2_ for magnetic hydrogels appeared at strains approximately 10^−2^ and clearly decreased with the carrageenan concentration. As γ_2_ corresponds to the onset strains where *G*″ starts to increase, it can be considered that γ_2_ is a characteristic strain at which the physical bond between carrageenan chains and/or the physical contact between magnetic particles through the bound layer of carrageenan start to destruct by the strain. At 500 mT, the γ_2_ for magnetic hydrogels clearly shifted to lower strains in a range of approximately 10^−4^. It can be considered that γ_2_ under the magnetic field is the characteristic strain at which the chains of magnetic particles start to be destructed by the strain.

The characteristic shear stresses σ_1_ and σ_2_ were explained by the following equations:

σ_1_ = *G*′_γ1_γ_1_

**Figure 5:**
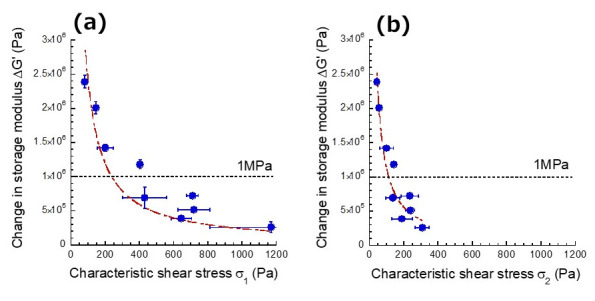
Relationship between change in storage modulus and characteristic stress (**a**) σ_1_ or (**b**) σ_2_ for magnetic hydrogels with various carrageenan concentrations (CI: 70 wt%).

σ_2_ = *G*′_γ2_γ_2_

where *G*′_γ1_ and *G*′_γ2_ represent the storage moduli at γ_1_ and γ_2_, respectively. Figure 5a shows the relationship between characteristic stress σ_1_ at γ_1_ and the change in storage modulus for magnetic hydrogels. It was seen that the change in storage modulus decreased with the characteristic stress. This strongly suggests that magnetic particles are hard to move within the carrageenan network with the increasing of σ_1_.

Figure 5b exhibits the relationship between characteristic stress σ_2_ at γ_2_ and the change in storage modulus for magnetic hydrogels. Similar to the behavior of σ_1_, the change in storage modulus decreased with the characteristic stress, suggesting that the movement of magnetic particles is depressed with the increasing of σ_2_. Thus, the characteristic stresses at γ_1_ and γ_2_ were distributed at 80–720 Pa and 40–310 Pa, respectively. The dotted lines in the figures appear to show the change in storage modulus with 1 MPa. This means that a giant magnetorheology higher than 1 MPa can be observed when the flowability of the gel-matrix is high, where the characteristic shear stresses satisfy a condition of τ_1_ < 240 Pa or τ_2_ < 110 Pa.

**Figure 6:**
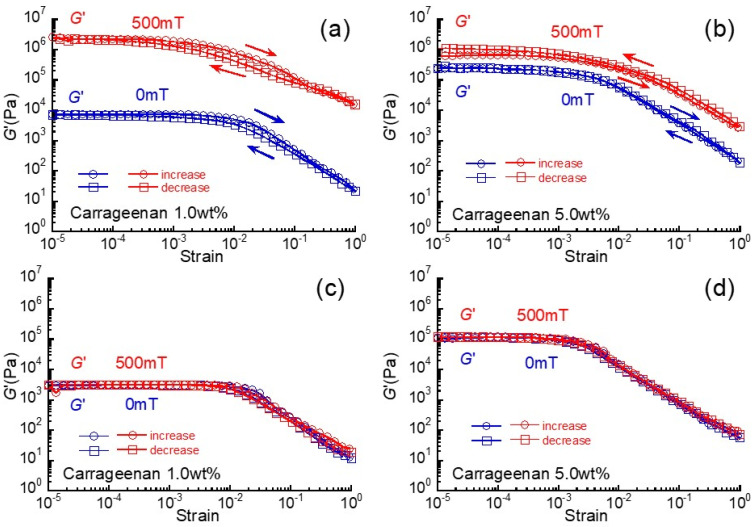
Hysteresis in the strain dependence of storage modulus for (**a**,**b**) magnetic hydrogels (CI: 70 wt%) and (**c**,**d**) carrageenan hydrogels without magnetic particles. (**a**,**c**) 1 wt% carrageenan, (**b**,**d**) 5 wt% carrageenan.

Figure 6 shows the hysteresis in the strain dependence of storage modulus at 0 and 500 mT for magnetic hydrogels and carrageenan hydrogels without magnetic particles. At 0 mT, there was no hysteresis in the storage modulus for both gels, regardless of the carrageenan concentration. As described in Figure 1, the carrageenan network was destructed by large strains higher than 10^−3^–10^−2^. However, the destructed network perfectly recovered to the original modulus after the strain was reduced. At 500 mT, it was observed for both magnetic hydrogels (1 wt% and 5 wt% carrageenan) that the storage modulus when reducing the strain was higher than that when increasing the strain. This behavior is typical, and has been observed for magnetic hydrogels, magnetic particles are highly aligned by both large strain and magnetic field. Our previous reports [23,25] using a pulsatile magnetic field showed that the storage modulus for magnetic hydrogels reversibly changes in response to the magnetic field at approximately of 100 times. Accordingly, this strongly indicates that the storage modulus recovers to the original value although the magnetic particle does not recover to the original distribution.

**Figure 7:**
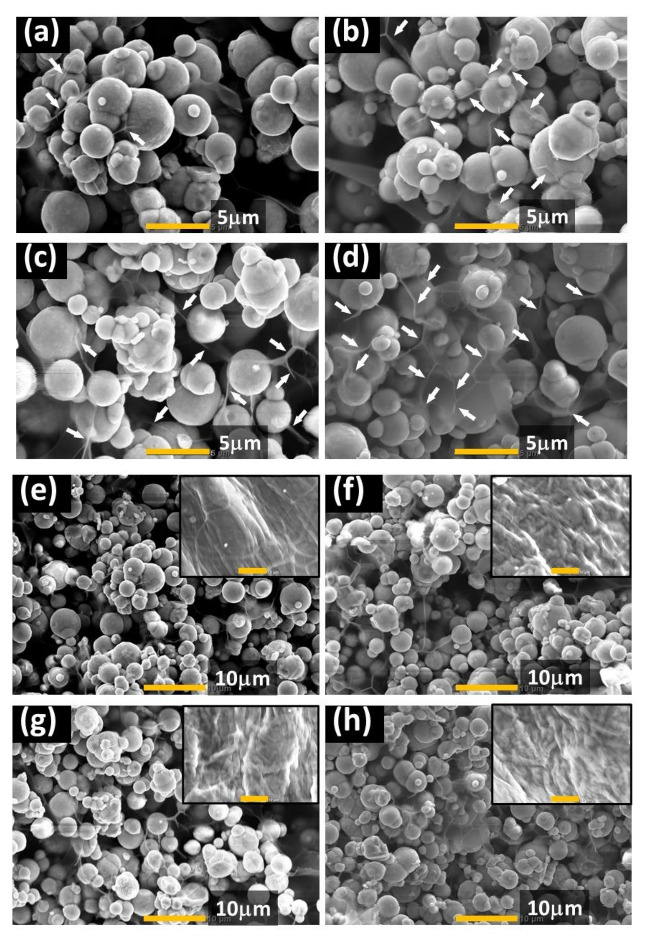
SEM photographs for 1 wt% carrageenan magnetic hydrogels taken at various conditions: (**a**,**e**) initial state before applying strain, (**b**,**f**) after applied large strain, (**c**,**g**) after applying a magnetic field of 500 mT, and (**d**,**h**) after applying large strain under a magnetic field of 500 mT. Inset: 1 wt% carrageenan hydrogels without magnetic particles. Magnification (**a**–**d**) ×5500 (scale bar = 5 μm) and (**e**–**h**) ×2400 (scale bar = 10 μm). Stretched fibers of carrageenan are indicated by arrows in (**a**–**d**).

Figure 7 indicates the SEM photographs for magnetic hydrogels taken at various experimental conditions in Figure 6. Note that all photographs were taken in the absence of a magnetic field. Figure 7a shows an image taken at initial state before applying large strains, which is the same as a magnetic hydrogel, as synthesized. Magnetic particles were randomly distributed in the matrix of carrageenan hydrogel, which agrees with our past studies [23,25]. Moreover, it is clear that magnetic particles were coated by carrageenan chains. It has been reported for fucan polysaccharide that there is a self-adhesion property between polysaccharides and magnetic particles [32]. Figure 7b presents a photo taken after applying large strains, which corresponds to a condition after carrying out one-cycle strain dependence at 0 mT. Short fibers of carrageenan seem to be increased by large strains. Figure 7c shows a photo taken after applying a magnetic field of 500 mT without applying shear. Figure 7d exhibits a photo taken after applying large strains under the magnetic field. Many short fibers that connected magnetic particles are also seen in these images. The insets of Figure 7e–h show the SEM photographs for carrageenan hydrogels without magnetic particles. Thick fibers of carrageenan with several microns were observed in these images, which is typically observed in polysaccharide hydrogels. Generally, the thick fibers are rarely seen in magnetic hydrogels and the long filaments of carrageenan are observed as indicated by arrows in Figure 7a–d. There exists a hydrogen bond between -OH groups in carrageenan and on the surface of the CI particle [33]. By applying the magnetic field, the polysaccharide chains of carrageenan would be stretched by the relative movement of magnetic particles as the chain is partially adhered on the surface of CI particles.

## 3. Conclusions

The relationship between the flowability of the gel-matrix on the magnetorheological response was investigated using magnetic hydrogels with various carrageenan concentrations. The change in storage modulus decreased inversely proportional to the carrageenan concentration, suggesting that magnetic particles are difficult to move in the gel-matrix. As a parameter indicating the flowability of magnetic particles, we proposed two kinds of characteristic stresses σ_1_ and σ_2_. It was cleared that the change in storage modulus decreased inversely proportional to these characterisctic stresses. Additionaly, it was found that the stress σ_1_ should be lower than 200–300 Pa to generate a modulus increase with an order of 1 MPa. SEM images revealed that the application of the magnetic field accelates fibering of carrageenan chains rather than the application of large strains. These insights could be useful for designing the polymer network of gels, enabling high mobility of magnetic particles.

## 4. Materials and Methods

### 4.1. Synthesis of Magnetic Hydrogels

The polymer matrix of magnetic hydrogels is κ-carrageenan of a polysaccharide (*M*_w_ = 857 kDa, CS-530, San-Ei Gen F.F.I., Osaka, Japan). Magnetic particles are carbonyl iron (CS Grade BASF SE., Ludwigshafen am Rhein, Germany). A pregel solution of the magnetic hydrogel was prepared by mixing 1 wt% carrageenan aqueous solution and magnetic particles at 100 °C using a vortex mixer for approximately 1 min. The pregel solution was poured into a mold made of two sheets of glasses and a silicone spacer and was cooled to 20 °C to obtain the magnetic hydrogel. The weight concentration of magnetic particles was kept at 70 wt%, e.g., magnetic particle of 4.79 g and 1 wt% carrageenan aqueous solution of 2.05 g. Carbonyl iron (CI) particle (CS Grade BASF SE., Ludwigshafen am Rhein, Germany) was used as a magnetic particle, which has a saturation magnetization of 190 emu/g and a remanent magnetization of 1.6 emu/g. The magnetization curve and the particle size distribution for the magnetic particle (same manufacturing lot number) were reported in previous papers [24,26]. The median diameter for magnetic particles was determined to be 7.0 ± 0.3 μm by a particle size analyzer (SALD-2200, Shimadzu Co. Ltd., Kyoto, Japan).

### 4.2. Rheological Measurements

The magnetic response of dynamic modulus for carrageenan hydrogels and magnetic hydrogels was measured by dynamic viscoelastic measurement using a rheometer (MCR301, Anton Paar Pty. Ltd., Graz, Austria) with an electromagnet system (PS-MRD) and a nonmagnetizable parallel plate (PP20/MRD) at 20 °C. The strain was varied from 10^−5^ to 1, while the frequency was constant at 1 Hz at all measurements. The direction of shear is perpendicular to the magnetic field. The sample was a disk 20 mm in diameter and 1.5 mm in thickness. The data of storage modulus shown in Figure 2, Figure 3 and Figure 5 were determined by averaging over three different samples in a single batch.

### 4.3. SEM Observations

Scanning electron microscope (SEM) observation was carried out using JCM-6000 Neoscope (JEOL Ltd. Tokyo, Japan) with an accelerating voltage of 15 kV without Au coating. Magnetic hydrogels with a size of φ20 mm × t1 mm were dried by freeze-drying for 5 h without applying a magnetic field. The cross section of these dried gels was observed. Apparent shrinkage was not observed for the magnetic gel owing to the freeze-drying (less than 3% in length).

## Data Availability

The data presented in this study are available in article.
